# Electronic Device Use before Bedtime and Sleep Quality among University Students

**DOI:** 10.3390/healthcare9091091

**Published:** 2021-08-24

**Authors:** Hue Thi Pham, Hsiao-Ling Chuang, Ching-Pyng Kuo, Tzu-Pei Yeh, Wen-Chun Liao

**Affiliations:** 1 Department of Nursing, Duy Tan University, Da Nang 550000, Vietnam; phamthihue3@duytan.edu.vn; 2 School of Nursing, Chung Shan Medical University, Taichung 40201, Taiwan; ling0911@csmu.edu.tw (H.-L.C.); pyng@csmu.edu.tw (C.-P.K.); 3 School of Nursing, China Medical University, Taichung 406404, Taiwan; 4 Department of Nursing, China Medical University Hospital, Taichung 404332, Taiwan; 5 Department of Nursing, Asian University, Taichung 41354, Taiwan

**Keywords:** sleep quality, electronic devices use, university students

## Abstract

Using electronic devices before bedtime impacts sleep quality and has become a major public health issue. This study aims to investigate the associations between electronic devices (EDs) use before bedtime and sleep quality in Vietnamese university students. A total of 369 university students from three departments were recruited. Participants completed self-report surveys, including demographic characteristics, lifestyle, ED-use behaviors, the Pittsburgh Sleep Quality Index, and the Center for Epidemiologic Studies Depression Scale. A total of 48.8% of the students experienced poor sleep quality, and 98.1% reported using at least one type of ED every day within two hours before bedtime. Smartphones are the most used devices (92.3%). ED usage within two hours before bedtime (*p* = 0.031), lack of exercise (*p* = 0.006), alcohol consumption (*p* = 0.025), and coffee intake after 4 pm (*p* = 0.018) were associated with poor sleep quality. ED use near bedtime for a duration longer than 30 min (*p* = 0.001) and depression (*p* < 0.001) were associated with poorer sleep quality among university students. ED use near bedtime more than 30 min was significantly associated with poorer sleep quality after adjusting depression status, exercise, and caffeine/alcohol intake in the latter part of the day. This study emphasizes the importance of adequate sleep and restriction of ED use near bedtime, which are necessary for better sleep in university students.

## 1. Introduction

Sleep is a biological need for human survival. Adequate sleep is crucial for a healthy and productive life. Sleep quality during the night influences our energy in the day. Evidence shows that adequate sleep can maintain physical and mental health [1]. Good quality sleep at the right time can also improve learning and memory [2]. For university students, adequate sleep may influent their academic performances, problem-solving skills, emotional status, and safety in life [1]. Although most people know that an adequate amount of and good quality sleep are related to some benefits in maintaining health, university students appear not to pay much attention to their sleep hygiene, and exhibit a lack of motivation towards establishing good sleep habits. According to the latest guidelines from the National Sleep Foundation, an optimal amount of sleep is 7–9 h for young adults; however, only 24% of university students sleep more than 8 h per day [3]. University students tend to neglect their sleep on weekdays [4], and over 40% experienced poor sleep quality, based on the Pittsburgh Sleep Quality Index (PSQI) measures survey [5,6,7]. Furthermore, 20% of students reported that they had stayed awake all night at least once a month [7]. In developing countries such as Vietnam, 49.4% of students had poor sleep quality, with a mean sleep duration of 6.1 h [8].

Sleep is a periodic resting behavior which is synchronized in circadian rhythms [9]. Light is the strongest synchronizing zeitgeber for this circadian system [10]. Environmental light synchronizes the primary mammalian biological clock in the suprachiasmatic nuclei (SCN) to keep biological and psychological rhythms internally synchronized for optimum function [10]. Human follows the light cycle for daily activities such as sleep. Artificial illumination affects our rhythm in increasing alertness and cognitive performance [11] and also in suppressing sleep [12]. Higher illuminance of light exposure, especially blue-enriched light before bedtime, was associated with more alertness, delayed phase, less slow-wave sleep, and prolonged sleep latency [13,14,15].

Exposure to the light from screens in smartphones, computer, or tablets etc., which contain blue-enriched, short-length light, is similar to exposure under morning sunlight [16]; this condition should be avoided before bedtime. However, as a result of technological developments, the usage of electronic devices (EDs) including smartphones, computers, laptops, and tablets has significantly increased over the past decade globally. With numerous functions and easy access, EDs have become an important part of students’ daily lives. Students study and spend leisure time on EDs every day. The increasing time spent on ED use not only in daytime but also before bedtime should be noticed. ED usage near bedtime has become one of the foremost factors associated with sleep disturbance, in addition to various other factors such as caffeine consumption, exercise amount, tobacco smoking, alcohol consumption, prolonged working time, and depression. The proportion of the internet use in young Vietnamese adults was high in 15–19 years old (92%) and 20–29 years old (76%) [17]. The peak hours of smartphone and laptop use were near bedtime, from 8–10 pm [18]. It means that young adults were exposed on short-wavelength light before bedtime through using these EDs. The use of EDs at inappropriate times may result in melatonin suppression caused by the short-wavelength light emitted from the EDs’ screens [19], and may take individuals a longer time to fall asleep and reduce their total sleep duration [20,21].

Social media use is increasing continuously in developing countries. Approximately 81% of Vietnam’s young adults aged 18–29 prefer to use these EDs in accessing social networking sites [22]. Moreover, Vietnam is a developing country with a high prevalence (78.1%) of sleep problems among university students [23]. Therefore, it is necessary to investigate the associations between ED use and sleep quality in young Vietnamese adults. Most ED usage in sleep-related studies focused on children and adolescents, and consistent results were found between ED usage and poor sleep quality [24]. Young adults are more mature than children and adolescents, this means the impacts of ED use in young adults may be different from the adolescents. Only few researchers have investigated the relationship between young adults’ sleep quality and the overall using habits of smart phones [25,26], and only one study examined the influence of digital media use in the two hours before bedtime on sleep quality in university students [27]. Although light exposure before bedtime may have a greater impact on sleep, only 37% of studies have focused on the effects of pre-bedtime ED usage on sleep quality [24]. Furthermore, ED types and usage patterns are changing rapidly; thus, continuing to monitor this is necessitated. Except for ED-using time, ED-use behaviors such as screen mode adjustment, ED mode, and ED location while sleeping need further investigation. This study recruited university students between 18 and 25 years old in a developing country, and aims to gain an insight into their sleep quality and ED-use behaviors and evaluate the potential influences of ED use within two hours before bedtime on their sleep quality. Lifestyle behaviors such as exercise [28], caffeine intake [29], alcohol intake [30,31], tobacco smoking [32], and depression [1] are important factors associated with sleep quality, and were set as covariates in this study.

## 2. Materials and Methods

### 2.1. Study Design

A cross-sectional study with a convenient sampling method was used to recruit university students from January to April 2019. Students were recruited from the nursing department, economic law department, and the electronics department. The potential participants were approached during term time when they had class meetings. The researcher explained the study purpose and that it may take 10–15 min to complete the questionnaires anonymously. Those students who were willing and volunteered to participate in this study had to sign the informed consent form before they answered the questionnaires. The researcher instructed the participants put the paperback questionnaires in self-sealing envelopes and return them to the researcher. Students could withdraw from this survey at any time without any explanation.

### 2.2. Participants

**Figure 1:**
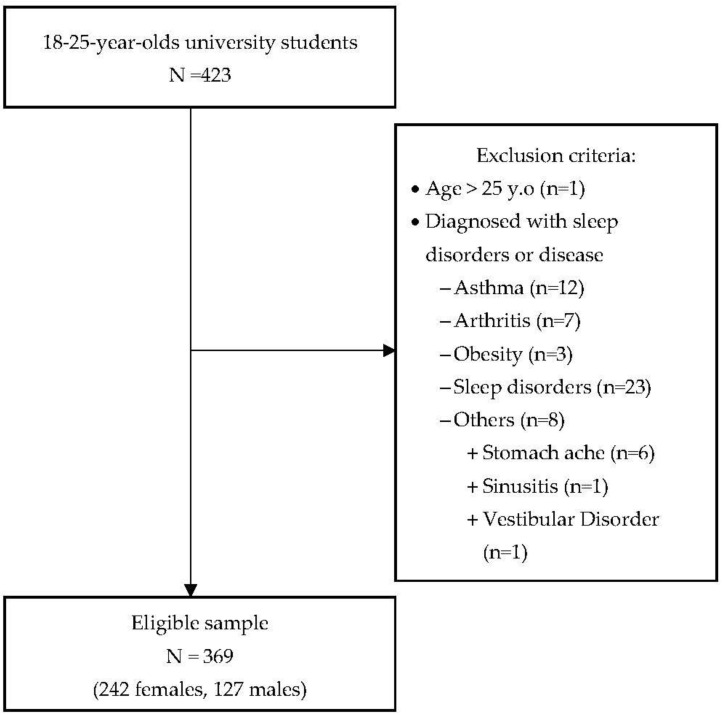
Flowchart of the participants’ selection process.

Students aged between 18 and 25 years old participated in this study (242 females and 127 males) (Figure 1). Those older than 25 years old (*n* = 1) or who had been diagnosed with chronic diseases or sleep disorders (*n* = 53) were excluded. This research was reviewed by the Institutional Review Board of the Duy Tan University, Danang, Vietnam (DTU-IRB 20190013), and permission was granted by the nursing, business law, and electronics departments. The students volunteered to participate in this study with informed consent.

### 2.3. Measures

The self-report questionnaires consisted of five parts: (1) sleep quality, (2) electronic devices (EDs) use, and covariates of (3) depression, (4) lifestyle behaviors, and (5) demographic characteristics.

Sleep quality

The students’ sleep quality was assessed by using the Vietnamese PSQI (PSQI-V) [33]. The PSQI has nineteen items in seven categories: sleep duration, sleep disturbance, sleep latency, daytime dysfunction, sleep efficiency, subjective sleep quality, and use of sleeping medication. It aims to assess students’ sleep quality in the previous month. Each component was scored from 0 to 3, with a total score between 0 and 21. Higher scores indicated poorer sleep quality. A score of greater than 5 indicates poor sleep, whereas a score of equal or less than 5 indicates good sleep quality [34]. The correlation coefficient of Vietnamese version with the original PSQI English version was 0.77, and the test–retest reliability coefficient was 0.79 [33]. The overall internal reliability of Cronbach’s alpha coefficient for the PSQI-V in this study was 0.65.

ED use

To assess ED-use behaviors, students were asked to indicate their ED-use habits (yes, no), the types of EDs used (smartphone, iPad/tablet, TV, laptop, computer, music device, game console), frequency of ED use (one to three times per month, once per week, several times per week, daily), duration of ED use within two hours before bedtime (15–30 min, 30 min to 1 h, 1–2 h, more than 2 h), screen light adjustment (yes, no), place(s) where they keep their EDs while sleeping (under the pillow, beside the bed, in the bedroom but further than five meters from the bed), and ED-use modes (silent, vibrate, normal, airplane) during sleep. The duration was the past month.

Depression

The brief Center for Epidemiologic Studies Depression Scale (CES-D), Vietnamese version [35], was used to measure depressive symptoms among university students. Participants reported their feelings or behaviors during the past month with 16 items using a 5-point Likert scale, ranging from 1 = Never to 5 = Always. A higher score indicates a higher level of depressive symptoms, with a total score ranging from 16 to 80 [35]. The total internal consistency (Cronbach’s alpha) of the brief CES-D Vietnamese version in this study was 0.83.

Lifestyle behaviors and demographic characteristics

Lifestyle behaviors, including consumption of caffeinated drinks (coffee, tea, milk tea, energy drinks, and soft drinks), alcohol consumption, smoking, and exercise in the past month, were collected. For consumption of caffeinated and alcohol drinks, the following questions were posed: “Did you consume any of these drinks after 4 pm in the past month?” Response options were “none,” “one to three times per month,” “once per week,” “a few times per week,” or “every day.” The smoking habit item was phrased as a “yes” or “no” question. The item about exercise duration was phrased as follows: “Did you exercise for 30 min at least three times per week in the past month?” Demographic characteristics data include age, sex, which year of study, department, and living status.

### 2.4. Data Analysis

Descriptive statistics were compiled to analyze the students’ demographic characteristics, lifestyle behaviors, depression, and ED use within two hours before bedtime. One-way analysis of variance (ANOVA) was used to examine the mean differences in the PSQI-V scores for the ED use, lifestyle behaviors, and characteristics variables. The strength of the association between the PSQI-V score (sleep quality) and the CES-D score (depression) was analyzed by using the Pearson’s correlation coefficient. Multiple linear regression was used to examine the association between ED use and sleep quality, while controlling for covariate factors. Data were analyzed using the Statistical Program for Social Sciences (SPSS) version 25.0 for Windows. Statistical significance was set at *p* < 0.05.

## 3. Results

A total of 423 students were recruited and 369 students were included in this study. Table 1 shows the demographic characteristics and lifestyle behaviors. Most students came from the nursing department (60.2%), followed by the economic law department (22.8%), and the electronics department (17.1%). Two-thirds of the students were female, more than 70% were sophomores or juniors with a mean age of 20.3 ± 2.5 years, and the majority of them lived with friends (45.5%) or family (37.4%). More than half (52.0%) drank all kinds of caffeinated drinks more than twice weekly, approximately one-third (36.0%) consumed alcoholic drinks, and a small proportion (4.3%) smoked. Only one-third (37.4%) had exercise habits (Table 1). CES-D depression scores ranged from 18 to 60, with a mean of 35.5 ± 8.8.

Students who consumed coffee and alcohol after 4 pm and did not exercise had poorer sleep quality (ANOVA *F* = 5.055–7.611, *p* < 0.05; Table 1). Sleep quality was significantly poorer among participants who drank alcohol or coffee after 4 pm compared with those who did not. Students who exercised at least three times per week during the past month had better sleep than those who did not. The Pearson correlation coefficient between sleep quality (PSQI-V score) and depression symptoms (CED-S score) was tested and showed that higher levels of depressive symptoms were correlated with poorer sleep quality (*r* = 0.359, *p* = 0.000).

### 3.1. Sleep Quality

Table 2 shows the university students’ sleep quality. Approximately half (48.8%) of students had poor sleep quality (PSQI-V scores > 5), and a large proportion (66.9%) went to bed after midnight. The majority students experienced sleep disturbances that occurred at least three times per week, namely “difficulty falling asleep within 30 min,” followed by “waking up in the middle of the night or early in the morning” and “having to get up to use bathroom.” The mean sleep duration was 6.39 ± 1.09 h, and only 43.6% of students reported that they slept for 7 h or more, which is recommended by the National Sleep Foundation for young adults.

### 3.2. ED-Use Behavior and Sleep Quality

ED use was shown in Table 3. Most patients (98.1%) used at least one type of ED within two hours before bedtime. Smartphones were the most frequently used devices (92.3%), followed by laptops (27.6%) and tablets (4.4%). Most (88.4%) of the students declared daily ED use, and only one-tenth (10.5%) used EDs “sometimes” within one week. More than half of the students used their devices for more than 1 h before bedtime. While using EDs before bedtime, 69.4% did not adjust the screen light (i.e., activating the night mode). Approximately half (50.1%) reported that they set their phones on normal mode and 69.4 % of them slept near their phone, including keeping their phone in bed (62.9%) or under a pillow (6.5%) while they were sleeping (Table 3). The results of a one-way ANOVA test for ED use and sleep quality (Table 3) showed that students who used any kind of EDs within two hours before bedtime had poorer sleep quality compared with those who did not. A longer duration of ED usage before bedtime was significantly associated with poorer sleep quality, especially in students who spent 1–2 h using EDs before bedtime. Students who placed their EDs five meters away from their bed reported better sleep quality than those who kept it in their bed or under their pillow. In addition, sleep quality was similar regardless of whether students adjusted the EDs screen’s light in the evening to reduce brightness and blue light exposure (Table 3).

### 3.3. Factors Associated with Sleep Quality

Multiple linear regression analysis was used to identify the factors influencing sleep quality in university students (Table 4). Factors were selected based on the results of the one-way ANOVA test and Pearson’s correlation analysis, including coffee intake, alcohol consumption, exercise, duration of ED use within two hours before bedtime (EDs_duration), ED location while sleeping (EDs_place), and depression. EDs_durations were categorized as no use, use less than 30 min, use 30 min to two hours, and use more than two hours; EDs_places were categorized as under pillow or in bed and_away from bed.

The results showed that EDs_use duration within two hours before bedtime and depression were predictors of university students’ sleep quality (*F* = 11.746, *p* < 0.000), with *R*^2^ = 0.207 (Table 4). Students who spent more than 30 min or more than two hours using EDs had poorer sleep compared with those who used less than 30 min, adjusted for coffee intake, alcohol intake, exercise, EDs_places, and depression. There was no difference between no use and less than 30 min of use. The PSQI-V scores increased by 0.094 units for each unit of depression (CES-D score) increased. Longer ED use duration and higher levels of depression were associated with poorer sleep quality among university students (Table 4).

## 4. Discussion

Students in this study reported a mean PSQI-V score of 5.7, which indicated a slightly poor sleep quality. Approximately half (48.8%) of the students had poor sleep quality, which was consistent with previous studies that reported a prevalence of 42.4–60% [5,6,7,26,36,37]. These findings were also similar to those of prior research conducted among Asian university students in Hong Kong (57.5%) and Taiwan (54.7%) [38,39], as well as central Vietnam (49.4%) [9]. The mean sleep duration of 6.39 (±1.09) indicated that many university students do not get enough sleep (7 h), in keeping with the National Sleep Foundation’s recommendation [40]. Not only do short sleep durations occur in Vietnam, they have also been reported in 26 other countries [41]. Furthermore, it has been reported that Southeast Asia has the shortest sleep duration among university students (mean nighttime sleep = 6.82 h) compared with other regions of the world (7.07 h) [41]. The prevalence of poor sleep quality is high among university students worldwide, making this issue a considerable health problem for young adults, since poor sleep adversely impacts physical and mental health, and may influence their studies as well.

Almost all students (98.1%) in this study accessed at least one type of electronic device within two hours before bedtime, 88.4% of them used EDs every day, and 53.1% spent more than one hour in current study. The phenomenon of heavy use in EDs in university students was similar to previous studies among young adults in that 72.9% participants reported daily smartphone use at bedtime [42] for more than one hour [27]. Reading and engaging with the computer for work and social communication simultaneously were the most common activities of ED use in university students, which was followed by listening to music [27]. These results indicated the EDs’ generous and inevitable use in terms of studying, social networking, and recreation. EDs have become essential instruments in daily modern life. How students can use them appropriately without addiction to EDs, which may affect their health and life, should be taken as an important issue when evaluating their healthy status.

Our findings demonstrated that using any kind of EDs within two hours before bedtime resulted in poorer sleep quality after controlling covariate factors (Table 4). Longer ED use duration before bedtime was also associated with poorer sleep quality. These results were consistent with a systematic review in adolescents who were aged from 6 to 19 years old, that a longer duration in screen ED use is associated with poor sleep quality in adolescents [20,23,26]. Although in this study, participants were older, aged 20 years and over, it is still notable that the association between ED use and poor sleep still was significant, which was consistent with findings among university students in developed countries [27,43] and developing countries [26]. The longer time spent on EDs, the greater decline in sleep duration and quality. However, these study results also showed that students who used EDs for less than 30 min within two hours before bedtime did not differ from those who did not use EDs. This may suggest that using EDs for less than 30 min before bedtime is acceptable and has no adverse effect on students’ sleep quality, whereas a previous study done in adults revealed a higher risk of having poor sleep quality in those who used smartphones at bedtime for more than 60 min [42]. Therefore, restricting time for ED use in the two hours before bedtime to 30 min is suggested in younger adults who are facing sleep problems.

Most EDs emit short-wavelength light (blue light), which can inhibit melatonin production, trigger acute alertness, reset circadian rhythms, and alter sleep time [13]; this mechanism may increase sleep onset latency and reduce sleep duration [24]. Only 30% of students in this study adjusted the ED screen’s light before bedtime (Table 3). Although our results showed no significant differences in sleep quality between adjusting and not adjusting screen light mode, avoiding exposure to blue light within 2–3 h before bedtime was still suggested. Vietnamese university students may not be aware of the effect of blue light from EDs enough. However, it seems quite difficult for university students to avoid using EDs before bedtime, especially in doing assignments and social activities. Another strategy that has been proposed is wearing blue light blocking glasses while using EDs before bedtime. A study involved 13 first-year undergraduate students to investigate the effect of a 2-week protocol in wearing blue light blocking glasses at night, and it found that the glasses can improve sleep quality and reduce sleep disturbances in young adults [44]. In addition, installing a blue light filter app for nighttime ED use seems practical for university students to reduce blue light exposure, and this strategy needs further investigation.

With the increasing use of high technology teaching methods with EDs, those of a younger generation, such as university students, may prefer to select online courses rather than in-classroom courses. The effect of ED use on sleep quality was recognized as a healthy issue which could influence their study performance. Interventions are needed to reduce the impact of ED use on sleep quality, such as delivering a sleep hygiene program to students. In the program, the harmful effects of using EDs near bedtime on diminished quality of sleep should be emphasized. Thirty minutes of ED use two hours before bedtime may be the maximum allowing time of bedtime ED usage. Some tips can be offered to minimize ED use near bedtime, for instance, filter app, smart phones using a time control app or blue-light blocking glass might be helpful. Setting up alarm clock one hour before bedtime may help student to remind themselves to turn off their EDs. Further work to examine the effectiveness of these strategies is necessary.

Several limitations should be considered when interpreting the results of this study. First, this was a cross-sectional study to investigate the association between ED-use behaviors and students’ sleep quality, but no causal relationships were tested among these factors. Second, a self-report method was used to collect the data within one month, which may cause recall bias. Future studies may combine other sleep measurements, such as a sleep diary or sleep trackers, to ensure the accuracy of the sleep data. Finally, the effect of blue light from EDs is considered one of the key underlying mechanisms in the relationship between ED use and sleep quality. The amount of blue light exposure from EDs must be examined in future studies.

## 5. Conclusions

Accompanied with the technology revolution, the use of EDs such as smartphones, computers, laptops, and tablets has increased significantly. ED use is inevitable and necessary. Longer ED use duration before bedtime is associated with poorer sleep quality. The prevalence of poor sleep quality among university students is high. Using EDs for less than 30 min before bedtime may be acceptable among university student in order to reach good sleep quality, but overuse should be avoided. How we can use EDs in a better way so as not to disturb sleep remains a global and crucial issue. Good sleep quality plays an important role in students’ health and well-being. Raising awareness about the importance of adequate sleep for health, how EDs could impact their sleep, and the imperative need to adjust their ED use near bedtime is necessary to improve their sleep quality.

## Figures and Tables

**Table 1:** ANOVA of demographic characteristics and lifestyle behaviors on sleep quality (PSQI-V Scores) (N = 369).

Factors	*n*	%	Mean	SD	*F*	*p*
Gender						
Female	242	65.6	5.87	2.64	2.536	0.112
Male	127	34.4	5.38	3.11		
Year of study						
Freshmen	75	20.3	5.65	2.78	1.166	0.322
Sophomore	155	42.0	5.45	2.73		
Junior	111	30.1	6.10	2.79		
Senior	28	7.6	5.61	3.39		
Department						
Nursing	222	60.2	5.70	2.62	0.108	0.898
Law	84	22.8	5.61	3.00		
Electronics	63	17.0	5.83	3.22		
Living status						
Family	168	45.5	5.31	2.80	2.395	0.093
Alone	138	37.4	6.16	3.06		
Friend(s)	63	17.1	5.85	2.70		
Caffeinated drink consumption ^a^						
No	177	48.0	5.42	2.86	3.420	0.065
Yes	192	52.0	5.96	2.75		
Coffee						
No	312	15.4	5.55	2.74	5.655	0.018
Yes	57	84.6	6.51	3.09		
Tea						
No	339	8.1	5.68	2.79	0.226	0.635
Yes	30	91.9	5.93	3.11		
Milk tea						
No	253	31.4	5.56	2.810	2.053	0.153
Yes	116	68.6	6.01	2.805		
Energy drinks						
No	331	10.3	5.66	2.81	0.484	0.487
Yes	38	89.7	6.00	2.85		
Soft drinks						
No	303	17.9	5.62	2.76	1.442	0.231
Yes	66	83.1	6.08	3.03		
Alcohol consumption ^b^						
No	236	64.0	5.45	2.60	5.055	0.025
Yes	133	36.0	6.14	3.11		
Smoking						
No	353	95.7	5.68	2.76	0.503	0.478
Yes	16	4.3	6.19	3.90		
Exercise ^c^						
No	231	62.6	6.01	2.61	7.611	0.006
Yes	138	37.4	5.18	3.07		

^a^ Determined by the response to “Did you usually consume any caffeinated drinks after 4 pm more than twice per week during the past month?” ^b^ Determined by the response to “Did you typically consume any alcohol after 4 pm in the past month?” ^c^ Determined by the response to “Did you exercise last month at least three times per week?”.

**Table 2:** Sleep Quality among University Students as Assessed by the PSQI-V (N = 369).

	*n*	%
Sleep quality (PSQI-V)	5.7 ± 2.8 (0–16)	
Good sleep (PSQI-V ≤ 5)	189	51.2
Poor sleep (PSQI-V > 5)	180	48.8
Subjective sleep quality		
Very good	61	16.5
Fairly good	239	64.8
Fairly bad	67	18.2
Very bad	2	0.5
Sleep latency		
<15 min	215	58.3
16–30 min	95	25.7
31–60 min	36	9.8
>60 min	23	6.2
Sleep disturbance ^a^		
Unable to fall asleep within 30 min	65	17.6
Waking up in the middle of the night or early in the morning	35	9.5
Having to get up to use the bathroom	27	7.3
Unable to breath comfortably	10	2.7
Coughing or snore loudly	14	3.8
Feeling too cold	28	7.6
Feeling too hot	18	4.9
Having bad dreams	17	4.6
Experiencing pain	13	3.5
Other reasons	19	5.1
Sleep duration	6.4 ± 1.1 (311) hrs	
>= 7 h	161	43.6
6–6.9 h	137	37.1
5–5.9 h	56	15.2
4–4.9 h	15	4.1
Habitual sleep efficiency (SE) ^b^		
>85%	241	65.3
75~84%	77	20.9
65~74%	43	11.7
<65%	8	2.2
Use of sleeping aid medication		
None during the past month	363	98.4
<once/week	4	1.1
1–2 times/week	2	0.5
>= 3 times/week	0	0
Daytime dysfunction		
Trouble staying awake while driving, eating meals, and/or engaging in social activity		
Not during the past month	209	56.6
<once/week	86	23.3
1–2 times/week	58	15.7
>= 3 times/week	16	4.3
Trouble with enthusiasm to get things done		
No problem	76	20.6
Slight problem	208	56.4
Somewhat problematic	79	21.4
Very problematic	6	1.6
Going to sleep after midnight	247	66.9

^a^ At least three times per week for one month. ^b^ SE = (total hours asleep)/(total hours in bed)*100.

**Table 3:** ANOVA of ED-use behaviors on sleep quality (PSQI-V Score) (N = 369).

Factors	N	%	Mean	SD	*F*	*p*
ED use						
No	7	1.9	3.43	1.40	4.696	0.031
Yes	362	98.1	5.74	2.82		
ED use frequency						
Daily	320	88.4	5.85	2.88	1.847	0.138
Sometimes/week	38	10.5	4.97	2.19		
Once/week	2	0.6	3.50	0.71		
1–3 times/month	2	0.6	6.00	2.83		
ED use duration						
No use (0)	7	1.9	3.43	1.397	9.842	0.000 ^a^
15–30 min (1)	63	17.1	4.38	2.03	(4) > (2) > (1) ^b^;	
30–60 min (2)	103	27.9	5.40	2.42	(4) > (0)	
1–2 h (3)	107	29.0	5.93	2.98	(3) > (1) ^b^; (3) > (0)	
>2 h (4)	89	24.1	6.88	3.06		
ED location while sleeping						
Under a pillow (1)	24	6.5	6.71	3.41	3.722	0.012
In bed (2)	232	62.9	5.91	2.80	(1) > (3) ^c^	
In the bedroom but 5 m or more from the bed (3)	106	28.7	5.08	2.61	(2) > (3) ^c^	
Outside the bedroom (4)	7	1.9	4.43	2.22		
Adjust ED’s screen display light						
No	110	30.4	5.91	2.72	2.884	0.090
Yes	252	69.6	5.36	3.01		
ED mode while sleeping						
Normal	185	50.1	5.55	2.64	0.164	0.921
Airplane	92	24.9	5.68	2.69		
Silent	49	13.3	5.95	3.24		
Vibrate	43	11.7	5.70	2.99		

*Note*. EDs = Electronic devices ^a^ Using Levene Statistics based on the homogeneity of variance tests *p*-value < 0.05. ^b^ Post hoc test by using Dunnett’s T3 test based on the significant homogeneity of variances. ^c^ Post hoc test by using the least significant difference test based on the homogeneity of variances assumed.

**Table 4:** Multiple linear regression analysis for sleep quality with related factors.

Variables	B	95% CI	T	*p*
Coffee ^a^	0.726	[−0.016, 1.467]	1.923	0.055
Alcohol ^b^	0.058	[−0.529, 0.646]	0.196	0.845
Exercise ^c^	−0.311	[−0.879, 0.256]	−1.079	0.281
EDs_place ^d^	−0.381	[−0.963, 0.201]	−1.289	0.198
Depression ^e^	0.095	[0.064, 0.127]	6.025	0.000
EDs_duration ^f^				
EDs_duration_less than 30 min	1.575	[−0.422, 3.573]	1.551	0.122
EDs_duration_30 min_2 h	2.588	[0.667, 4.510]	2.649	0.008
EDs_duration_more 2 h	3.343	[1.383, 5.304]	3.353	0.001

*R*^2^ = 0.207, *F* = 11.746, *p* = 0.000. Abbreviations: EDs = electronic devices. ^a^ Coffee: reference group = no coffee. ^b^ Alcohol: reference group = no alcohol. ^c^ Exercise: reference group = no exercise. ^d^ EDs_place: reference group = EDs kept close to the bed (under the pillow/in the bed); 1, EDs kept far from the bed (in the bedroom but at least 5 m from the bed). ^e^ Depression was measured using the CES-D. ^f^ EDs_duration: EDs usage duration variable, reference group = no use; 1 = less than 30 min; 2 = 30 min to 2 h; and 3 = more than 2 h; these variables were classified into dummy variables for analysis.
